# A cross-sectional study of partograph utilization as a decision making tool for referral of abnormal labour in primary health care facilities of Bangladesh

**DOI:** 10.1371/journal.pone.0203617

**Published:** 2018-09-06

**Authors:** Abdullah Nurus Salam Khan, Sk Masum Billah, Ishtiaq Mannan, Imteaz Ibne Mannan, Tahmina Begum, Marufa Aziz Khan, Munia Islam, S. M. Monirul Ahasan, Jebun Nessa Rahman, Joby George, Shams El Arifeen, Umme Salma Jahan Meena, Iftekhar Rashid, Joseph de Graft-Johnson

**Affiliations:** 1 Maternal and Child Health Division, International Centre for Diarrhoeal Disease Research, Bangladesh (icddr,b), Dhaka, Bangladesh; 2 Save the Children, Dhaka, Bangladesh; 3 Health Systems and Population Studies Division, International Centre for Diarrhoeal Disease Research, Bangladesh (icddr,b), Dhaka, Bangladesh; 4 Jhpiego Corporation, Dhaka, Bangladesh; 5 USAID Bangladesh, Dhaka, Bangladesh; 6 USAID Bangladesh, Dhaka, Bangladesh; 7 Department of Global Health, Save the Children, Ellicott City, Maryland, United States of America; World Health Organization, SWITZERLAND

## Abstract

**Background:**

In Bangladesh, female paramedics known as Family Welfare Visitors (FWVs), conduct normal deliveries in first-level primary care facilities, or Union Health and Family Welfare Centres (UH&FWC). Utilization of partographs allow for early identification of abnormal labour and referral for advanced care to Emergency Obstetric Care (EmOC) facilities. A systematic assessment of the quality of partograph utilization in clinical-decision making will contribute to understanding the use of the tool by health workers.

**Methods:**

In 2013, the USAID supported MaMoni HSS project, led in country by Save the Children, trained FWVs on the use of partographs in five UH&FWCs in Habiganj district. As part of the follow-up after training, intrapartum case record forms, accompanying partographs, and referral registers for all obstetric cases managed in these five facilities from July 2013 to June 2014 were reviewed. Partographs were reviewed to identify abnormal labour cases based on pre-defined indications. All referred cases were ascertained from the case records in the referral registers. Five health workers were interviewed to assess their knowledge, attitude and experience in partograph use and to explore the challenges for referral decision making associated with the tool.

**Results:**

A total of 1,198 deliveries were managed at the study sites, of which 663 presented with cervical dilatation of 8 cm or less. Partographs were initiated in 98% of these cases. Indication of abnormal labour was found in 71 partographs (11%) and among them, only 1 was referred to a higher-level facility. Foetal heart rate and cervical dilatation were appropriately recorded in 61% and 70% of the partographs, respectively. Interviews with health workers revealed poor interpretation of referral indications from the partographs. Limited accessibility to the nearest EmOC facility, inadequate time for referral, and non-compliance to referral by clients were identified by the interviewed health workers as the key barriers for referral decision making.

**Conclusions:**

Supporting the health workers at first-level primary care facilities to better interpret and act on partograph data in a timely manner, and strengthening the referral systems are needed to ensure that women in labour receive the prompt quality care they and their babies require to survive.

## Introduction

A partograph is a simple, low-cost monitoring tool for intrapartum care recommended by the World Health Organization (WHO) which has the potential to identify obstetric complications by graphically presenting the critical events of labour progression, including the condition of both the woman and the foetus [1]. It also has impact on improving the quality of intrapartum care, maternal health and birth outcomes [2, 3]. A prospective multicentre trial conducted in South-East Asia by the WHO showed significant reduction in prolonged labour, need for labour augmentation and caesarean section, and number of intrapartum stillbirths where partographs were used along with appropriate labour management guidelines [4]. The effectiveness of partograph use in improving the birth outcome could be limited as suggest by a Cochrane review [5]. Especially in low and middle income countries with high maternal and neonatal mortality, this inexpensive tool is not used accurately or as intended during intrapartum care [6–9]. Although the Cochrane review could not recommend partograph for routine use based on the studies included in their review, it does leave the decision to include partograph as part of standard labour care to individual countries [5]. Bangladesh is one of the countries that has decided to make partograph use part of its standard for labour care [10].

Bangladesh is among the ten countries contributing to the major share (59%) of global maternal deaths [11] and two-thirds of its maternal deaths are due to direct obstetric causes, including prolonged and obstructed labour [12]. The recent five year strategic plan, Health, Population and Nutrition Sector Program 2017–2022 (HPNSP), has therefore focused on identifying complications during labour and appropriate referral to Emergency Obstetric Care (EmOC) health facilities as an effective way to reduce the maternal and neonatal mortality [13]. Campbell et al. (2006), in the Lancet Maternal Survival Series, also prioritized delivering in primary care facilities with improved access to referral centres [14]. Union Health and Family Welfare Centres (UH&FWC) are the first level of primary care centres in the government health system of Bangladesh. These centres are staffed by a single health worker, trained to offer basic obstetric services to a population of 24,000–30,000 in a union (lowest administrative unit) [15]. Utilization of partograph at this level of facility is vital to guide the single health worker to identify abnormal labour and to implement the appropriate management, including prompt referral to higher level facilities equipped with EmOC signal functions at district hospitals, selected sub-district hospitals, Maternal and Child Welfare Centres (MCWC) and Medical College hospitals [16]. A needs assessment undertaken in Bangladesh found that partographs are only used in 3% of all deliveries conducted in health facilities [17]. The lack of partograph use might result in poor identification of intrapartum complication and delayed intervention [18]. A systematic review of partograph suggests its potential to trigger the referral decision of critical obstetric cases by health workers [19]. However, there are limited studies on the use of partographs as a referral decision making tool by health workers in primary level care facilities [20, 21].

The study aimed to address two research questions—i) whether partographs are utilized for referral decision making by the single health worker at the first level of health care in Bangladesh-UH&FWCs and ii) what factors are associated with health workers’ decision making, such as their knowledge and attitude towards the use of partographs and their experience regarding the referral of women with abnormal labour. The findings from this study will guide the programme designers and policy makers to adopt pragmatic solutions for improving the use of partographs for better management of labour and prompt care for the survival of mothers and their babies.

## Materials and methods

### Study setting

The study was part of a programme learning activity of USAID supported MaMoni Health Systems Strengthening (MaMoni HSS) project, led in country by Save the Children. The MaMoni HSS project supported the strengthening of selected UH&FWCs in Habiganj district of Bangladesh to ensure round-the-clock services to conduct child birth along with essential newborn care services [22]. Habiganj is situated on the north-eastern part of Bangladesh and covers a large area of wetland with seasonal variation of road networks. The study selected UH&FWCs in Kakailsheo, Doulatpur, Murakuri, Shibpasha, and Khagaura unions purposively because each of them provided round-the-clock services to conduct childbirth at the time of data collection. Each UH&FWC is staffed by a single health worker, or Family Welfare Visitor (FWV), with 18 months of pre-service training and are entitled to provide routine antenatal care, delivery care and postnatal care [15]. In five of these UH&FWCs, MaMoni HSS project recruited female paramedics with 36 months of pre-service training to support the FWVs in providing the same package of health services for mothers and their newborn. The FWVs were supervised by health managers posted at sub-district hospital and paramedics were supervised by project recruited field quality assurance officer. As part of the implementation of standards-based management of labour, the project also provided both cadres of these health workers with 15 days of in-service training on partograph and knowledge and skills on appropriate management of all the three stages of labour. The training also included the indications that required immediate referral to EmOC equipped facilities. The partograph used in the training material was WHO modified partograph which excludes latent phase of labour monitoring and the instructions were written in English on partograph paper. The training material was developed by the MaMoni HSS programme in consultation with Obstetrical and Gynaecological Society of Bangladesh. The referral conditions included foetal distress, pre-eclampsia, and prolonged and obstructed labour and the health workers were instructed to refer women upon finding any of these conditions. The designated first level referral facility for UH&FWCs is the sub-district hospital, known as Upazila Health Complex (UHC). During the study period, none of the UHCs in Habiganj district offered comprehensive EmOC services due to the absence of the mandated surgeon-anaesthetist pairs and lack of blood transfusion facilities. The only place in the district for referral of complicated obstetric cases was the Habiganj district hospital, a secondary level facility. The project also ensured availability of partograph paper in each health facility. Routine follow-up monitoring and mentoring visits were made jointly by the project staff and the line supervisors from government to ensure the utilization of partographs during normal deliveries conducted at the selected facilities. Table 1 shows means of transport and travel time to the nearest functional comprehensive EmOC centre from the five selected UH&FWCs.

**Table 1:** Type of transportation and travel time to the nearest comprehensive EmOC referral facility.

Name of UH&FWC^1^	Nearest comprehensive EmOC^2^ referral facility	Means of transport^4^	Travel time^4^
Daulatpur	Habiganj district hospital^3^	Three-wheeler	2.5 hours
Kakailsheo		Engine boat (wet season) Three-wheeler, then engine boat (dry season)	3.0 hours 2.5 hours
Khagaura		Three-wheeler	40 minutes
Murakuri		Three-wheeler	1.0 hour
Shibpasha		Three-wheeler	1.0 hour

^1^ Union Health and Family Welfare Centre

^2^ Provision for emergency caesarean section, blood transfusion, newborn resuscitation

^3^ Secondary level facility

^4^ Information obtained from MaMoni HSS project data

### Study design

A cross-sectional study design using multiple data collection methods was used. Data was extracted from the following documents in all five selected UH&FWCs: 1) intrapartum case record forms, 2) partographs, and 3) referral registers for all pregnant women who reported or were admitted in labour from July 1, 2013 to June 30, 2014. Intrapartum case recording forms were only completed when the pregnant woman was admitted at the facility for childbirth after initial examination. Key informant in-depth interviews with the health workers (i.e. FWVs and paramedics) were conducted. The interviews explored the knowledge and attitude of health workers towards utilization of partographs and their experience regarding use of partographs for decision making on referrals.

Table 2 presents the summary of all data collection methods and related objectives of the study. The number of cases and partographs reviewed per UH&FWC were proportionate to the caseload for delivery care services at those health facilities.

**Table 2:** Specific objectives, selection of samples and proposed methods.

Objective	Sample selection	Sample size	Method	Tool
Determination of obstetric status of pregnant women on arrival	All cases admitted in the selected facilities for childbirth during the specified time period	1198	Record review of intrapartum case recording form	Structured data extraction form
Determination of presence of indication for referral and partograph completeness	All cases admitted with cervical dilatation of 8 cm or less having a partograph	648	Record review of partograph	
Identification of reasons for referral	All cases referred from the selected facility for obstetric reasons	350 (without admission) 5 (admitted in labour)	Record review of referral register	
Exploration of health workers’ knowledge, attitude and experience in partograph use and challenges for partograph based decision making	Health workers providing round-the-clock services to conduct childbirth in the selected facilities	5	In-depth interview	Semi-structured interview guideline

### Data collection and storage

#### Quantitative data extraction

Intrapartum case record forms, partographs and referral registers were reviewed for all cases attending the selected facilities for childbirth during the specified time period. Regarding sample size estimation a decision was made to undertake a census of all childbirth cases occurring in the five facilities for the specified one year period after completion of staff readiness to use the partograph. Readiness included the staff training, supervision and mentoring and provision of necessary supplies including the paper partograph forms. One year was regarded as an adequate period to show any effect that partograph use might have on staff labour care practices. All of the reviewed partographs had instructions written in English. Three separate data extraction tools were developed based on the recording format of the government’s birth and referral register books, and the WHO modified partograph [23], and were reviewed by the obstetricians of research team (JNR and TB). Next, the data extraction forms were optimized for android based platforms by the team’s programme developers and checks were put in the programming to ensure data quality.

Four research assistants were recruited for data extraction and received a three-day orientation led by the obstetricians of the research team. In addition to leading the orientation, the obstetricians also reviewed the data extraction tools. The training focused on orienting the research assistants with case recording and reporting formats for maternal health services in government health facilities of Bangladesh. This was followed by an interactive training session on the ‘modified WHO partograph’. This part of the training focused primarily on orienting the research assistants with the different components of partographs, interpretation of the recording and completing the data extraction forms accordingly. The training was facilitated using the WHO partograph training manual for midwives (23) and the labour management protocol adopted in Bangladesh. The participants were also trained by the programme developers on using the data entry software on tablet phones. After the training sessions, the research assistants were individually tested for correctness of data extraction using sample partographs and evaluated by the obstetricians. The research assistants were re-oriented on the topic until they could enter information with a minimum of 95% accuracy.

Information about the obstetric history, including maternal age, gestational age, parity and findings of initial clinical assessment on arrival at the facility including status of membrane, and cervical dilatation, were obtained from the intrapartum case records. Obstetric history and initial examination findings were missing for the women who were referred immediately after initial assessment because they were not provided the intrapartum case record forms as they were not admitted. Their information was recorded only in the referral register. A review of records from referral registers also ascertained which of the obstetric cases were actually referred. Each obstetric case was also checked for the accompanying partograph. The partographs were reviewed for the plotting of different foetal and maternal components. The entire data extraction was conducted electronically using a tablet phone (Samsung Galaxy Tab 3.0). Each obstetric case was given a software generated unique ID. Based on this ID, data from three sources of records were one-to-one matched instantaneously in the software programme. The matching also confirmed the referral status of each obstetric case. Data from the individual data collectors was uploaded immediately to a central server and consolidated into one dataset.

#### Qualitative in-depth interviews

The in-depth interview guidelines were developed after analysing the extracted data. The quantitative findings guided the conceptualization and finalization of the major domains for in-depth exploration. The major domains of the interviews included health worker’s attitude towards the use of partographs, knowledge about partograph components and how to record them correctly, identification of abnormal labour from completed partographs and their management, and reasons for referral or missed referral. In each facility, an FWV who provides round-the-clock services, was selected for key informant interviews. In cases where the FWV was unavailable, a project supported paramedic was interviewed in their place. A total of five health workers, including one FWV and four paramedics, one from each of the selected UH&FWCs, were interviewed by a designated member of the study team. The interviewer did not take part in quantitative data extraction.

### Ethical clearance

The study protocol was reviewed by Jhpiego Corporation’s IRB determination committee and ethical approval was waived for the in-depth interview of the health workers as key informants since no personal identifier information was collected. The data extraction from government registers and partographs was only conducted upon receiving government approval and did not require collecting any personal identifiers.

### Data analysis

Background characteristics of pregnant women such as age, parity, dilatation of cervix on arrival at UH&FWC, status of membrane, place of childbirth are presented using frequency distributions.

Identification of abnormal labour and referred case: After the data extraction, information charted in the partographs was examined to identify what abnormal data on labour had been recorded. For identification of foetal or maternal abnormalities during labour, four conditions were specified as abnormal labour, namely, foetal distress, prolonged labour, obstructed labour and pre-eclampsia. National protocol for labour management also specify these conditions as indication for referral from first level facility to EmOC equipped primary level facility or higher [10]. A number of partograph-based indicators for these conditions were defined (Table 3) following the WHO guidelines [24]. A partograph was identified as having indication of abnormal labour during analysis if it had any of these indicators recorded.

**Table 3:** Definition of abnormal obstetric conditions using partograph information.

Abnormal Obstetric condition	Definition based on partograph plot
Foetal distress	• Foetal heart rate (FHR) less than 120/minute or more than 160/min for at least two observations • Meconium stained liquor
Prolonged labour	• Graph line for the cervical dilatation on right side to the alert line or reach action line
Obstructed labour	• Moderate or severe uterine contraction, along with any of the following ○Descent of the foetal head at plateau phase, or ○“+++” (severe) moulding of head
Pre-eclampsia	• Systolic blood pressure (BP) more than 140 mmHg or diastolic BP more than 90 mmHg, and presence of albumin in urine

Identification of referral status: All the reviewed partographs were matched with the referral register one-to-one to identify the referred cases and reasons for their referral. The referred cases which did not have an accompanying partograph were not included in further analysis. Birth outcomes for newborns, including neonatal death or stillbirth, were also obtained from the records. Referral reasons that were not related to pregnancy, such as general illness of mother, or postnatal reasons such as postpartum haemorrhage and retained placenta or neonatal conditions such as birth asphyxia, low birth weight, stillbirth and neonatal death, were considered beyond the scope of partograph based indication. Such cases were not considered as ‘referred cases’ during analysis.

Completeness of partograph: Only those cases who were received for childbirth in the facility with active stage of labour with cervical dilatation of 8 cm or less, were considered for analysis of partograph based data. Literature suggests that cases with cervical dilatation of more than 8 cm are near or at full dilatation, and hence do not require completion of a partograph as its use does not contribute to clinical decision making [20]. To assess the quality of partograph use in terms of completeness in charting, a simple descriptive statistical analysis was conducted. A component was considered correctly recorded if there were at least two plotting at the time interval suggested by the WHO and in other similar studies looking at the quality of partograph completeness [18, 25]. The appropriate time interval was ‘half hourly’ for monitoring of foetal heart rate (FHR), uterine contraction, and maternal pulse, ‘two hourly’ for monitoring of temperature and urine output, ‘four hourly’ for monitoring of state of liquor, moulding of foetal skull, cervical dilatation, descent of foetal head, and maternal blood pressure. The completeness of partograph components were compared between partographs with and without indications of abnormal labour using equality of proportions test (two proportions t-test). P-value less than 0.05 was considered significant to state two proportions were different. All quantitative data were analysed using Stata/SE 13.0.

In-depth interviews of the health workers: After completing the interviews, the audio recordings were transcribed verbatim and the transcription and field notes were translated into English. Data familiarization, a priori coding, and sub coding were done based on the research questions. Inductive codes were also generated to address the research questions and capture the concepts better. A sequential explanatory strategy was adopted to explain the quantitative findings through qualitative interviews i.e. findings from the partographs and records review led to developing qualitative guidelines.

## Results

### Background characteristics of pregnant women

**Fig 1:**
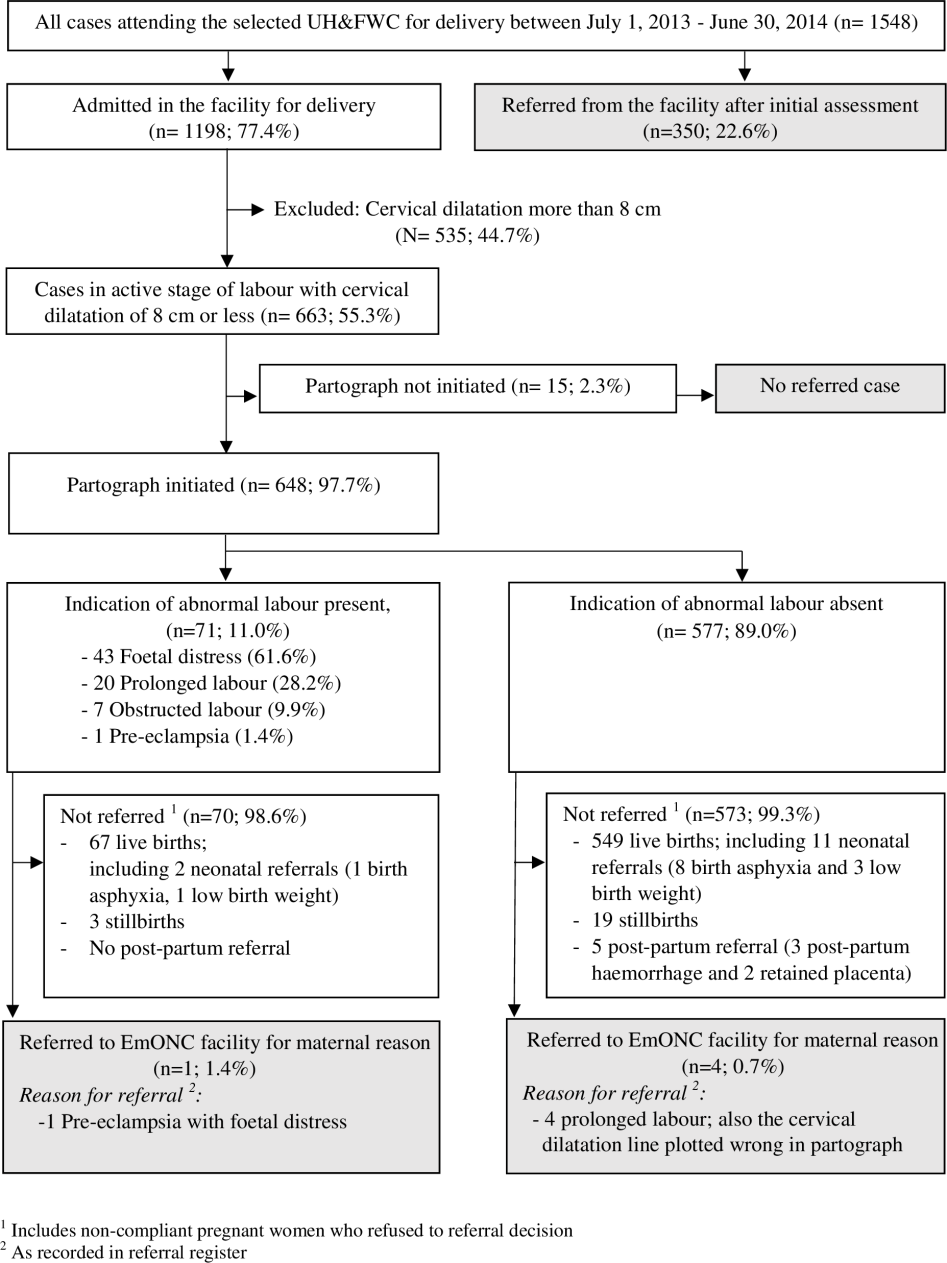
Flow diagram showing selection of cases with partograph, indication of abnormal labour in partograph and referred case with and without partograph indication of abnormality.

A total of 1,548 women in labour visited at the five study UH&FWCs during the specified one year period and only 1,198 women (77%) were admitted in the facility for childbirth after initial examination (Fig 1). The remaining 23% were referred to higher level facilities after initial assessment without admitting them for conducting childbirth. Table 4 shows the distribution of women admitted in the five health facilities by place of childbirth, age, parity, and dilatation of cervix and status of membrane on arrival at UH&FWC. The majority of the women delivered at Shibpasha and Kakailsheo UH&FWCs at 28% and 25%, respectively. Khagaura UH&FWC recorded the lowest percentage of women. The majority of pregnant women (78%) were aged between 20 to 29 years and overall, 34% were nullipara (first pregnancy). Over 55% of the women presented with cervical dilatation of 8 cm or less, and 85% presented with a ruptured membrane.

**Table 4:** Background characteristics of study population.

Background characteristics	% of pregnant women
Total, [n = 1198]	100.0
Place of childbirth	
Shibpasha	28.2
Kakailsheo	25.2
Murakuri	20.9
Doulatpur	19.4
Khagaura	6.3
Age of the pregnant women (completed year)	
Mean ± SD	24.9 ± 4.5
15–19	3.6
20–24	42.2
25–29	35.9
30 and above	17.1
No information recorded	1.3
Parity	
Nullipara (first pregnancy)	33.6
Multi-para	65.9
No information recorded	1.5
Dilatation of cervix on arrival	
8 cm or less	55.3
More than 8 cm	44.7
Status of membrane at admission	
Ruptured	84.7
Intact	5.6
No information recorded	9.7

### Presence of indication of abnormal labour in partograph and referral

The study revealed that partographs were used for 648 (98%) of the women with cervical dilatation of 8 cm or less (Fig 1). Seventy-one (11%) of the women who had partographs had at least one indication of abnormal labour based on the pre-defined criteria. Of these 71 abnormal partographs, foetal distress was the most commonly identified indication (62%), followed by prolonged labour (28%). Of the 648 women with completed partographs available, five were identified as referred to the EmOC facility due to abnormal labour conditions and only one of them had the indication recorded appropriately in the partograph. The remaining four cases were referred due to prolonged labour as reported in the referral register. However, the indication was not charted correctly in the accompanying partographs for these four cases.

### Quality of partograph recording

Out of 648 partographs reviewed for their quality of recording (Table 5), 61% had foetal heart rate (FHR) recorded, 68% had descent of foetal head and 18% had moulding of foetal skull recorded. The cervical dilatation line was plotted correctly in 70% of all partographs and in 82% of the partographs where any abnormality was indicated. The least and the most recorded data elements were maternal pulse (3%) and urine analysis for protein (92%), respectively. Blood pressure was recorded in two-thirds of all partographs. Partographs indicating abnormal labour had a significantly higher proportions (p-value<0.05) of recording for foetal heart rate, moulding of foetal skull, and cervical dilatation than those without indication of abnormal labour.

**Table 5:** Appropriate recording of partograph components for all women admitted for childbirth at the study health facilities.

Components of partograph	Appropriate time interval for assessing	% appropriately recorded in partograph			P-value ^a^
		All with 8 cm and below [n = 648]	Among those Indicated abnormal labour [n = 71]	Indication absent [n = 577]	
Foetal components					
Foetal heart rate^†^	Half hourly	61.0	71.8	59.6	**0.046**
Descent of foetal head	Four hourly	68.5	67.6	68.6	0.861
Moulding of foetal skull^†^	Four hourly	18.5	29.6	17.2	**0.011**
Maternal components					
State of liquor^†^	Four hourly	64.4	47.9	66.4	**0.002**
Cervical dilatation^†^	Four hourly	70.1	81.7	68.6	**0.023**
Degree of uterine contraction	Half hourly	77.8	73.2	78.3	0.329
Blood pressure	Half hourly	67.6	67.6	67.6	0.998
Pulse	Half hourly	3.2	0.0	3.6	-
Temperature	Two hourly	32.4	38.0	31.7	0.283
Urinary protein (albumin)	Two hourly	91.7	95.8	91.2	0.184
Amount of urine^†^	Two hourly	35.2	16.9	37.4	**0.001**

^a^ P-value for equality of proportion

^†^ P-value less than 0.05 and difference between proportions is significant

### Partograph use: Health workers’ knowledge and attitude

All of the five health workers stated that partographs helped them understand labour progression and identify risk factors during childbirth. The main perceived advantage was that it could document and present all vital information in one place. Especially in cases of referral, the reason was documented in the partograph.

One respondent said, *“If I had not recorded the findings intermittently*, *how could I compare the current status [with previous findings]*? *I may not identify critical conditions*. *My memory may not work properly*.*”*

Another respondent mentioned, *“An accident can happen any time*. *If I refer a mother*, *the reason for referral will be recorded in the partograph*. *This also makes us more accountable in case of referral*. *It’s for my safety as well as the safety of the patient*.*”*

The usual practice for completing the partographs was to record the findings first on a partograph-printed white board and to copy the plotting onto paper partographs after completion of the childbirth. When more than one patient was due for giving birth, this became a challenge for maintaining individual partographs at the same time.

One respondent described the scenario, “*There is only one board*, *and we keep the record of the second mother’s condition in a plain paper…then we copy that on a paper partograph using pencil which allows correction in case we make any mistakes while copying”*

They mentioned about the lack of knowledge on different components of the partograph. When asked to name the different components, all five health workers could mention about plotting the progress of cervical dilatation and the degree of uterine contraction in a partograph. But monitoring of foetal heart rate, degree of moulding and descent of head were mentioned by only two of them. Lack of confidence on plotting cervical dilatation, uterine contraction and descent of head were also mentioned by the health workers.

One health worker said, *“I am not much confident in using partograph*. *I know I make mistakes*, *but I still fill it up*. *I start partograph at 4 cm dilatation*, *but it is often difficult to identify the exact measure of (cervical) dilatation*.*”*

Three of the respondents mentioned about their difficulties in understanding the partograph since it was instructed in English and two mentioned about their difficulties in choosing the correct symbol for plotting a certain component.

One said, *“If there were Bangla instructions beside each component of partograph it would have been helpful for us to understand even if we forget [how to fill up the component]*.*”*

All five key informants mentioned that periodic but comprehensive in-service training would be helpful for them to utilize the partograph properly. They also mentioned the need for a practical demonstration, especially regarding the measurement of cervical dilatation. The health workers also expressed the need for frequent on-the-job feedback from the expert clinicians to identify their mistakes in partograph.

One stated, *“All of our partographs are checked by officer [non-technical programme staff]*, *but they only check if there is a partograph present for each delivery*, *and enquire for shortage of supply*. *They ask questions when there is some missing information in partograph and we explain the reason [to them]*.*”*

### Partograph based referral decision making: Health workers’ knowledge and experience

The in-depth interviews revealed the gap in knowledge in using partograph information for decision making. The interpretations and clinical importance of the partograph components were poorly understood by the health workers. Three out of the five health workers mentioned that they would refer a woman if her labour continued for more than 12 hours. Other indicators, such as foetal heart rate and meconium stained liquor, were rarely mentioned. None of the respondents reported that referral decisions could be made based on the degree of uterine contraction and moulding of foetal head. Two of the respondents asked for clear guidance on the management of identified labour abnormalities in partographs. However, they could identify pregnancy complications including prolonged labour pain, history of high blood pressure, convulsion, antepartum bleeding, and malpresentation from obstetric history and initial assessment. They also believed that they did not have to provide referrals because they felt competent and capable of managing cases of intrapartum complications including prolonged and obstructed labour based on their experience in handling complicated cases at UH&FWCs. Fear of referred women delivering in transit to the referral hospital was mentioned as a barrier for health workers wanting to comply with the management protocol for women with partograph determined indicator for referral. One respondent mentioned, *“If I refer a critical patient (in labour) and she delivers on the way*, *my performance will be seriously questioned*. *We will never find another woman coming to my facility for delivery*.*”*

The health workers also stated that the majority of the women coming to their facilities for childbirth resist going to a higher level facility. The reported reasons included cost of transportation and fear of giving birth to the child in transit. Two health workers mentioned that the time available for referral was often inadequate, as they would have already waited for too long at home before bringing the mother to the health facility. Two of the respondents stated the scenario as this:

“*The baby was in the mouth of cervix. We did not have any time to refer or maintain the partograph. We needed to attend the mother to conduct the delivery*”.

“*In case of mothers who are in critical condition but refuse to go to the referral facility, we obtain consent from them and take the risk of conducting the delivery at the facility*”.

The health workers reported on their inability to transfer abnormal labour cases to the designated first point of referral (sub-district hospital) due to their non-functional EmOC status, and referred to the Habiganj district hospital instead. In most of these types of cases, the challenge was to transfer the women from a peripheral to a central level hospital as the road networks were under-developed and the mode of transportation varied with seasonal changes.

One respondent mentioned, “*Upazila (sub-district) hospitals cannot manage obstructed labour*, *so we think about mother’s condition and refer to Sadar (district) directly with enough time at hand*”.

## Discussion

The study shows high usage of partographs at 98% for women in the active stage of labour at the first level of primary care facilities (UH&FWC) in the low resource settings of Habiganj district, Bangladesh. Yet low referral of abnormal labour indicated by partographs at only 1%, underscores the fact that increased utilization of partographs alone, without the implementation of standard guidelines for labour management and capacity improvements, does not suffice its use as a decision making tool [26–30]. The study also identifies a number of critical barriers to partograph based referral decision making. These include, incompleteness of the partograph and poor knowledge of health workers on partograph derived referral indications. In addition, lack of availability of EmOC services at first point of referral, poor accessibility to the referral facility, inadequate time for referral compliance due to late presentation at facility, and refusal of referral by women and family members indirectly influence the decision making of the primary care providers.

A decision for referral is initiated after the identification of abnormalities, thereby necessitating the correct use and skills required for partograph plotting [31, 32]. Our study shows that partographs without any indication of abnormal labour had significantly lower proportions of recording for foetal heart rate, moulding of head, and cervical dilatation than those showing indication of abnormality. This indicates that an incomplete and poorly charted partograph failed to instruct the health workers to take evidence-based decisions and increased the risk of handling complicated obstetric cases. This is further underscored by the fact that in our study the stillbirth rate among those with abnormal partographs was higher than those with normal partographs (4.2% of 71 abnormal and 3.3% of 577 normal partographs). Although it is worth noting that the number of stillbirths was too small to compare with statistical significance and the information on birth outcome was not adequately recorded in health facility registers to distinguish between fresh and macerated stillbirth. Several studies that looked at the quality of partograph completion also reported a wide range of sub-optimal recording of components pertinent to decision making [8, 18, 33, 34]. Sub-optimal documentation with limited utilization of partographs for decision making negates the observed increased completion of partographs. If health workers do not use the information recorded on partographs to inform evidence-based decision making, the care provided to the woman and foetus does not improve.

All of the health workers included in the study voiced their positive attitude towards utilizing partographs for monitoring and documentation of labour, although they had limited knowledge on partograph-based decision making. This finding is consistent with several studies reporting the persistent gap in knowledge about different sections of the partograph and their relevant role in labour management [7, 35–37]. In addition, the accuracy of the plotting remained unchecked during periodic checking by non-technical programme staff. As reported by the health workers, they were unable to provide expert feedback and only checked if the partographs were documented for all cases. A trial conducted in Indonesia not only showed high partograph usage (92%), but also ensured referral of almost two-thirds of identified prolonged labours following the implementation of obstetric care interventions in those facilities [38]. The intervention included on-the-job training of midwives and weekly supervision by obstetricians who would ensure the correct filling of partographs. Therefore, the pre-service training of the primary care providers (FWVs) should be accompanied by continuous education, refresher trainings and periodic supervision by specially trained nurses with midwifery skills or by the newly created midwives from higher level facilities [20, 38]. Innovative training approaches such as using the WHO e-learning tool for partographs can be considered, which was found feasible among midwifery students in Nairobi [39].

Several other contextual factors also played a vital role in referral decision making including remoteness of the facility and refusal of referral by the women and the family. Inconvenient accessibility to the nearest EmOC centre acted as a barrier for the pregnant woman and family to move to the district hospital even when the referral decisions were made by the health workers. Out of the five facilities, two were very remote with travel times to the nearest EmOC facility more than 2 hours. These facilities also lacked functional road networks during the dry season and had accessibility with engine boats during the wet season. This is a characteristic of wetland areas in north-eastern parts of Bangladesh [40]. A study conducted in the same wetland area also found that increased travel time to EmOC facilities resulted in lower rate of care seeking for institutional birth [41]. This also explains the resistant nature of the women and families to the referral advice. Given this context, the health workers rely more on women’s obstetric history, clinical assessment and overall health condition to make pragmatic decisions rather than utilizing partographs as the only guiding tool for referral decision making. Overall, this resulted in non-referral and the handling of many more critical cases at UH&FWCs, even when the partograph suggested a referral. Other studies also found that health workers’ competence to manage complications were taking priority over standard protocol and health workers were resistant to refer the critical cases identified by partographs to achieve a sense of professional competence in handling the abnormal labour [20, 38]. Development and implementation of standard guidelines for referral of abnormal labour with context-specific reinforcement may reduce reliance on health workers competency and ensure quality of intra-partum care [42]. At the same time, encouraging the health workers to follow the standard protocol with system reinforcement such as provision of in-service training with adequate supply of partographs and periodic checking for documentation and quality assurance [7, 26, 43] are required to improve ‘buy in’ of the health workers [44, 45]. For effective utilization of partographs and partograph-based decision making, health worker’s knowledge advancement through refreshers trainings, including practical demonstration, supportive supervision and on-site partograph audits by trained supervisors should also be prioritized. Strengthening the EmOC services in the nearest referral facilities along with improved transportation and referral tracking would further enable the workers to implement their decisions in cases of obstetric emergency.

In our study sites, the practice of using the white-board partograph and then transferring the plotting on to paper partograph was a problem when multiple clients were in labour at single-provider run UH&FWCs. This also raises the possibility that the reason for filling in the paper partograph in retrospect was to be able to be sure that it was consistent with the outcome, and concerns about the reliability of retrospectively entered partograph recordings. Few studies reported of retrospective documentation of partograph with bureaucratic priority following a government or non-government interventions which undermine the purpose of the partograph [43, 46]. At low resource settings where unavailability of paper partographs was reported to be a potential barrier [6, 27, 35, 47], introduction of electronic partographs in tablet or smart phones within routine health system could be explored. In Bangladesh, the government has already started distributing the tablet-phones to UH&FWCs in Habiganj district for recording the service statistics under the e-MIS (Electronic-Management Information System) initiative [48]. Altogether, this may serve as a platform for implementing paperless partograph use which could be incorporated with the referral algorithm, an interactive training instrument for the health workers and a tool for real-time remote consultation based on partograph findings with internet access [49]. Further research on innovative approaches on supporting the primary level health workers to correctly complete and interpret the information and comply with standard operating procedures for identified abnormal partographs, is required in resource-limited settings. There is also a need to ensure the referral systems and linkages to the well-established referral centres to enable prompt compliance with referrals and reduce the fear of women giving birth in transit. Appropriate counselling of the pregnant woman and her family by the health workers and awareness raising by the community health workers could successfully increase the referral compliance as seen in similar settings of Bangladesh [50].

### Limitations

The study had several limitations. First, the study did not include direct observation of labour management and only conducted retrospective review of completed partographs which might not depict the true quality of partograph completion. Second, we did not extract the data on partograph utilization prior to the project intervention as the research question was to identify to what extent a partograph is being utilized for decision making. Therefore, to see if there was any change in partograph use after supply of partographs, training and additional monitoring falls beyond the scope of this paper. Third, we considered at least two plotting to identify progress line for cervical dilatation and descent of foetal head in the partograph. However, repeated plotting would depend on the time between admission and giving birth and some women may have given birth before the next examination was due and the variables especially the ones requiring examination in every four hours could not have been measured again. This might underestimate the completion status of these components in partograph. Fourth, five health workers were interviewed, of which only one was a government FWV and four were project recruited paramedics. It is possible that performance of the two cadres could differ due to difference in duration of their pre-service training (FWV received 18 months and paramedics received 36 months of training). However, both of these cadres provided same obstetric services in UH&FWCs, received same package of training by MaMoni HSS project on labour progress monitoring and two of these cadres normally work together instead of running shifts. So information provided by 1 of the 2 adequately represents the knowledge and practices for that facility and is less likely to be biased due to their sample size difference. Although interviewing a larger number of health workers from diverse areas of the district could have identified more aspects on the use of partograph. Fifth, exploration of client’s perspective regarding non-compliance to referral decisions would have uncovered more about the context where the health workers are more inclined to handle complicated cases. Last, the study was conducted in the first level of primary care facilities that are supported by the project, and hence, the findings may not be generalizable to all of the facilities in the same tier. However, the circumstantial barriers of partograph-based referral decision making might be the same for other similar areas in the country.

## Conclusions

The study finds an increased use of partographs by the health workers for documentation purposes rather than using it as a guiding tool for identification of abnormal labour and appropriate referral and decision making on referral is dependent on more than correct interpretation of plotting on partographs. Poor knowledge about partographs and inadequate referral systems result in unduly reliance on health workers’ competence to manage complicated labours. It is essential to address provider attitude such as over-confidence in handling abnormal childbirth that should be referred. Guidance and associated skills set to complete the birthing process even if the partograph indicate an abnormality is needed for selected cases where the pregnant woman is likely to give birth before arriving at the next level of care. The findings direct maternal health programmes to consider the potential administrative and contextual factors for successful implementation of the interventions for improved quality of intrapartum care.
